# Supplementary material for: Activating hidden signals by mimicking cryptic sites in a synthetic extracellular matrix
Source: Nat Commun. 2023 Jun 19;14:3635. doi: 10.1038/s41467-023-39349-w (PMC10279755; doi:10.1038/s41467-023-39349-w)
Supplement: Supplementary file 1 — Supporting information [file 41467_2023_39349_MOESM1_ESM.pdf]

Supplementary Information

Activating hidden signals by mimicking cryptic sites in a synthetic extracellular matrix

Yumeng Zhu,[1#] Yulia Shmidov,[2#‡] Elizabeth A. Harris,[3] Michelle H. Theus,[3,4] Ronit Bitton[2,5*] and John B. Matson[1,4*]

[1]Department of Colhemistry and Macromolecules Innovation Institute, Virginia Tech, Blacksburg, Virginia, USA.

[2]Department of Chemical Engineering, Ben-Gurion University of the Negev, Beer-Sheva, Israel.

[3]Department of Biomedical Sciences and Pathobiology, Virginia Tech, Blacksburg, VA, USA.

[4]Center for Engineered Health, Virginia Tech, Blacksburg, VA, USA

[5]Ilse Katz Institute for Nanoscale Science and Technology, Ben-Gurion University of the Negev, Beer-Sheva, Israel.

*Corresponding author. Emails: jbmatson@vt.edu rbitton@bgu.ac.il

‡Present address: Department of Biomedical Engineering, Duke University; Durham, North Carolina, USA

# These authors contributed equally to this work

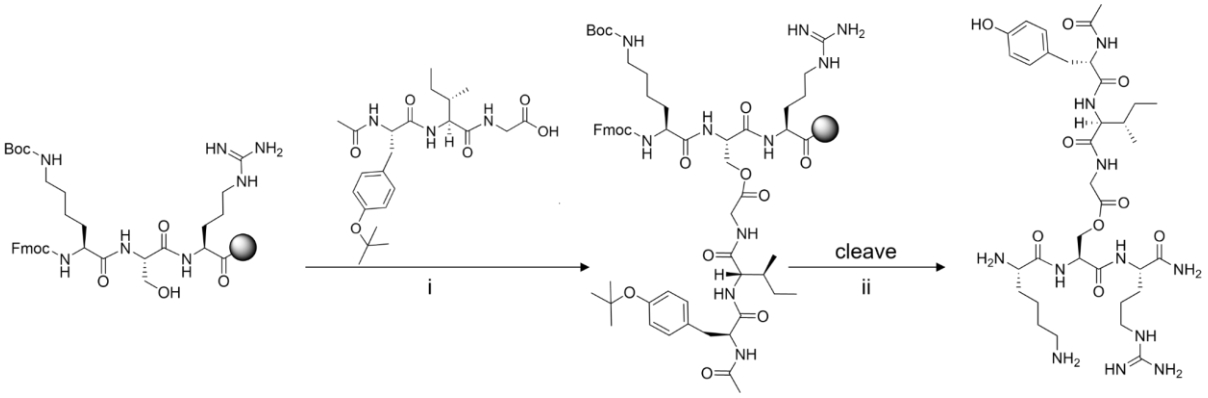

Fmoc-KSR Fmoc-KS$_{YIG}$R-resin KS$_{YIG}$R

Supplementary Fig. 1. Synthetic route to switch peptide KS$_{YIG}$R. Reaction conditions: i) DIC/DMAP, THF, rt, 4 h; ii) TFA/TIPS/H_2O (95%/2.5%/2.5%), rt, 3 h.

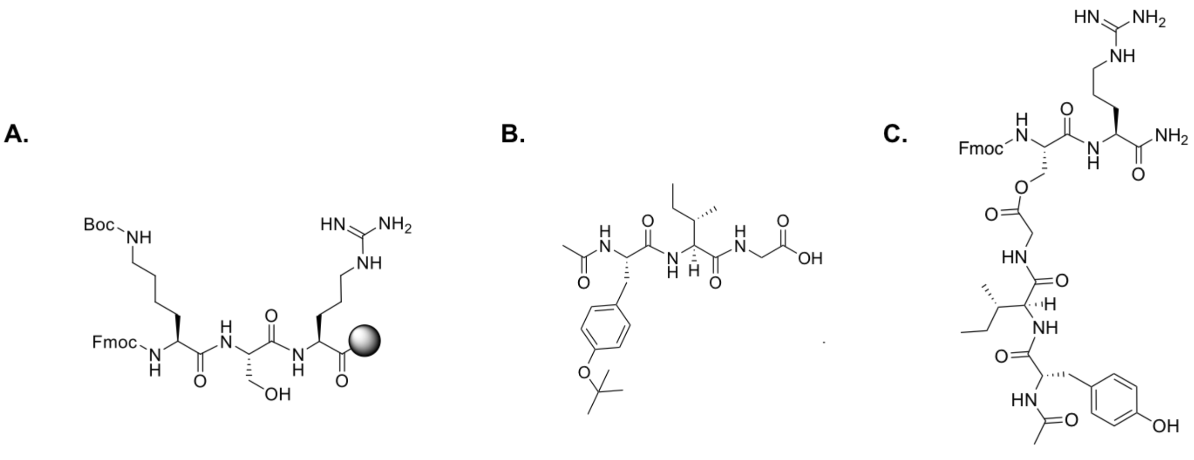

Fmoc-KSR Ac-YIG-OH Fmoc-S$_{YIG}$R

Supplementary Fig. 2. Chemical structures of (**A**) Fmoc-KSR on resin; (**B**) Ac-YIG-OH and; (**C**) Fmoc-S$_{YIG}$R

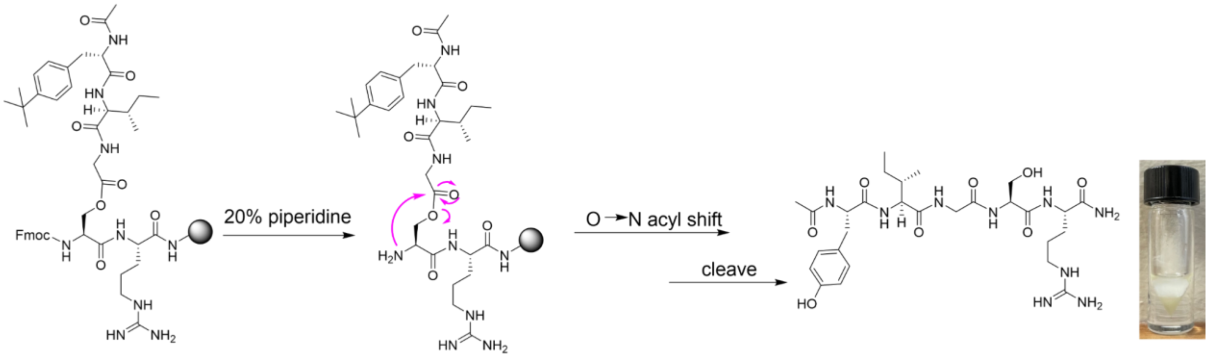

Fmoc-S$_{YIG}$R **intermediate peptide** **functional peptide**

Supplementary Fig. 3. **Structure of Fmoc-S$_{YIG}$R and conversion into intermediate peptide and functional peptide YIGSR**. Image shows Kaiser test result after piperidine treatment confirming the lack of free amines.

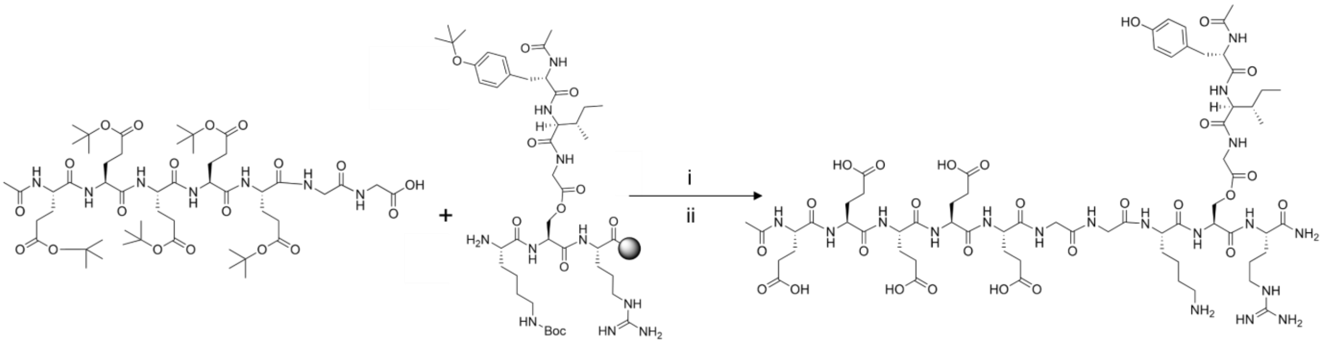

Ac-E5G2-COOH **KS$_{YIG}$R-resin** **Ac-E5G2-KS$_{YIG}$R**

Supplementary Fig. 4. Synthetic route to Ac-E$_5$G$_2$-KS$_{YIG}$R. Reaction conditions: i) pyBOP (1.1 eq), HOBt (1.1 eq), DIEA (2.2 eq), DMF, 75 ºC, 4 h; ii) TFA/TIPS/H_2O (95%/2.5%/2.5%), rt, 3 h.

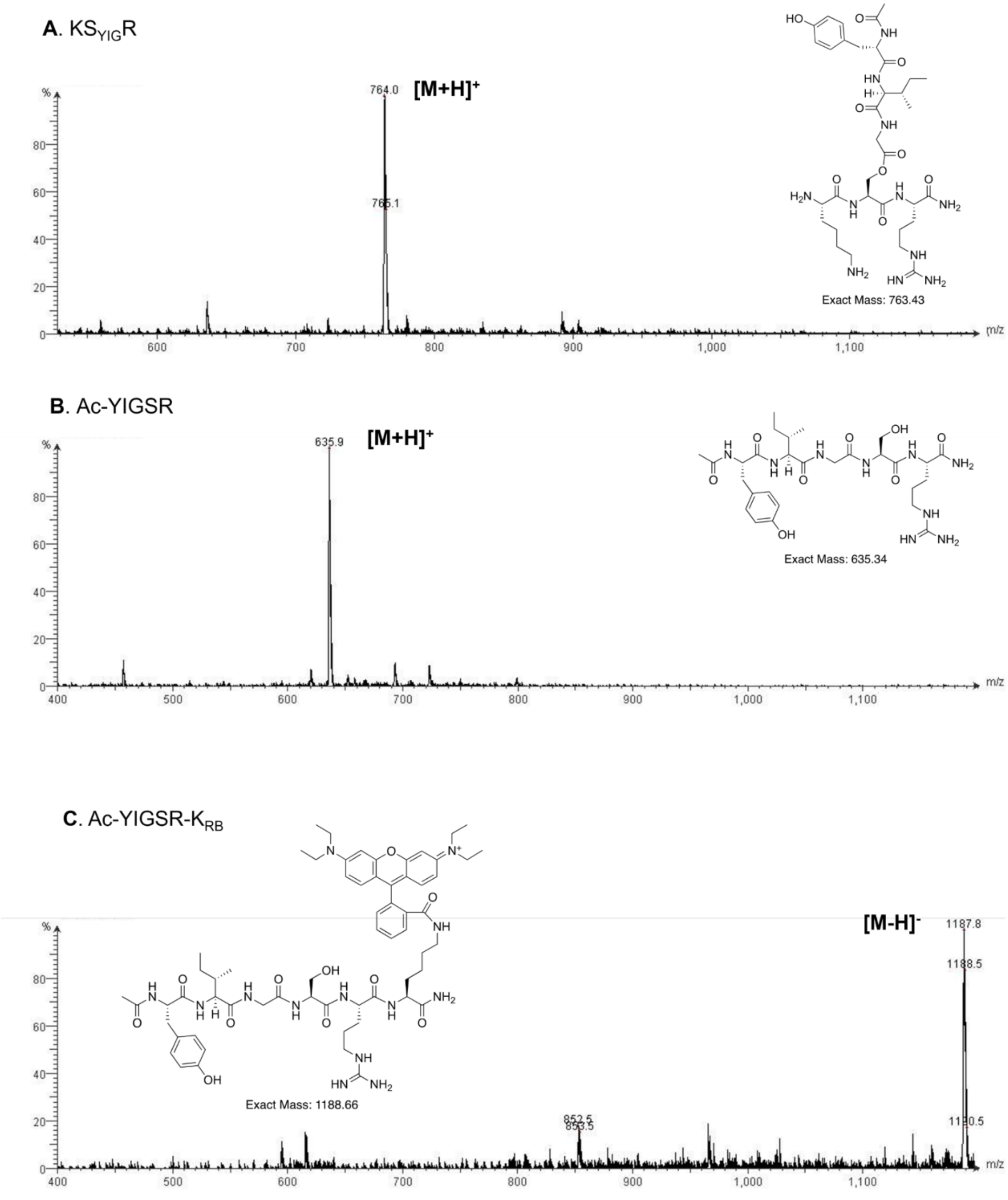

Supplementary Fig. 5. ESI mass spectra of (**A**) $KS_{YIG}R$; (**B**)Ac-YIGSR; and (**C**) Ac-YIGSR-K_{RB}.

A. Ac-E5G2-KS$_{YIG}$R

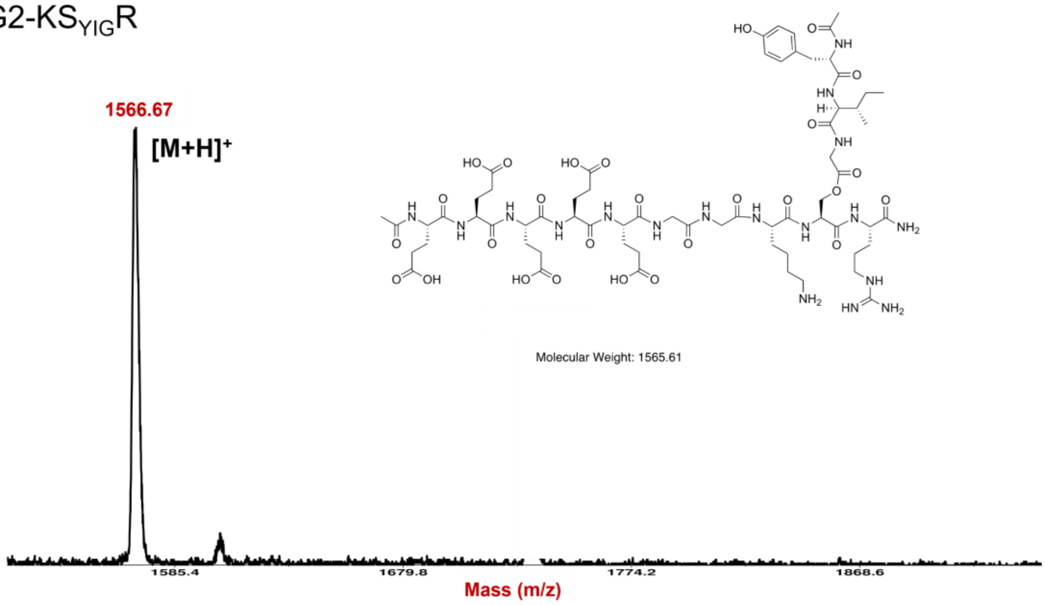

B. Ac-E5G2-KS$_{YIG}$R-K$_{RB}$

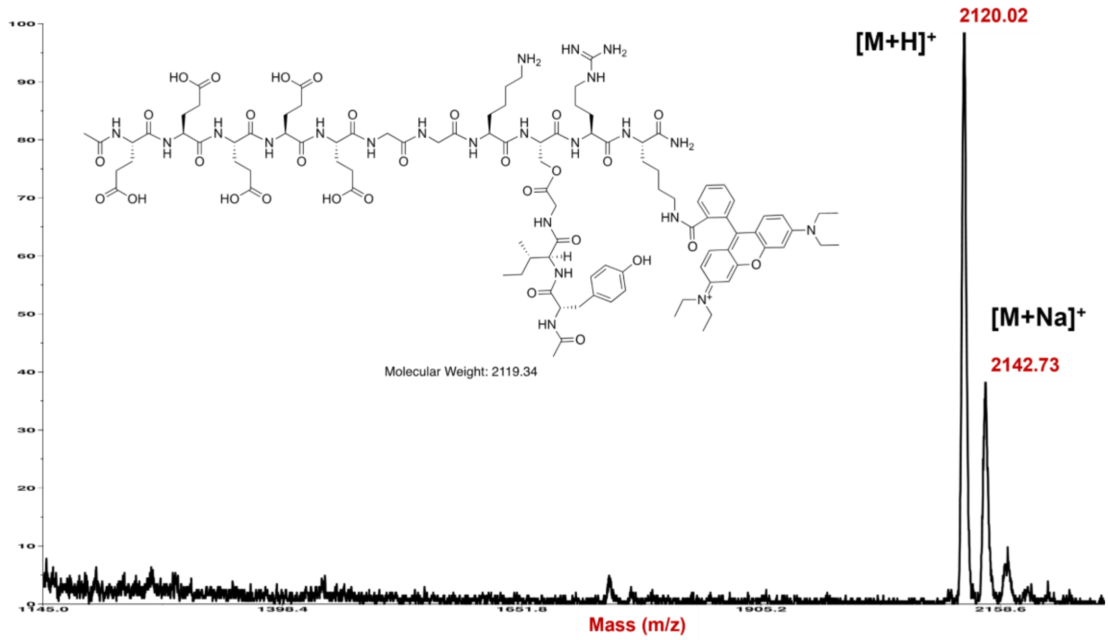

C. Ac-K_{YIG}-R-K_{RB}

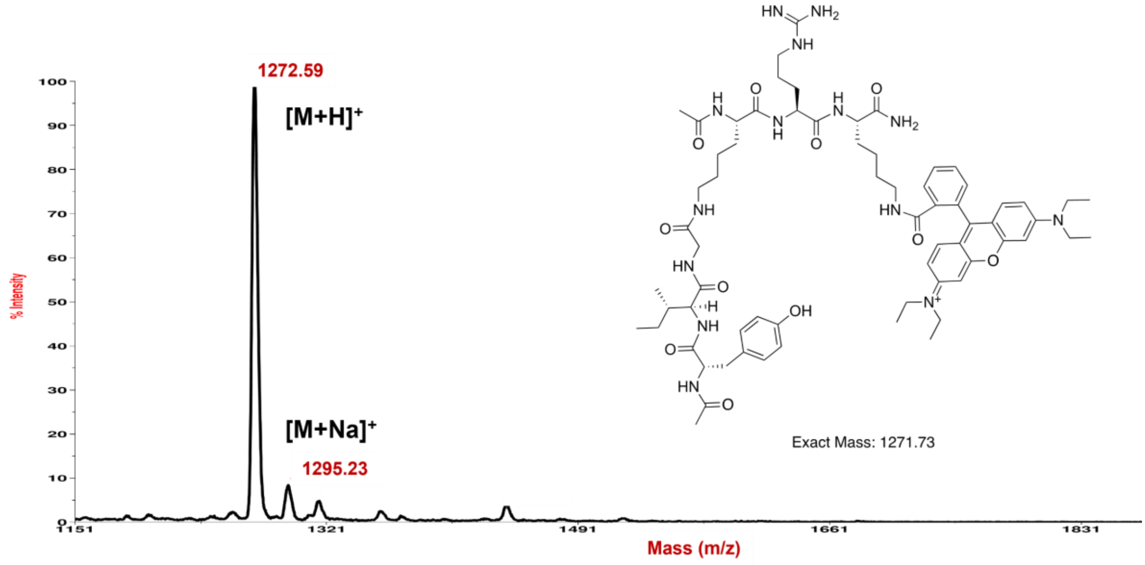

Supplementary Fig. 6. MALDI-TOF mass spectra of (**A**) Ac-E_5G_2-$KS_{YIG}R$; (**B**) Ac-E_5G_2-$KS_{YIG}R$-K_{RB}; and (**C**) Ac-$K_{YIG}R$-K_{RB}.

A. $KS_{YIG}RK$

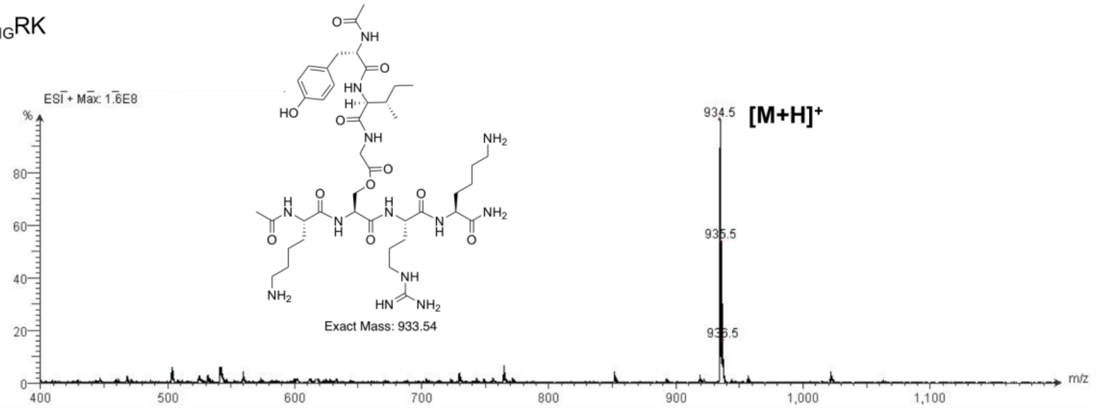

B. YIGSRK

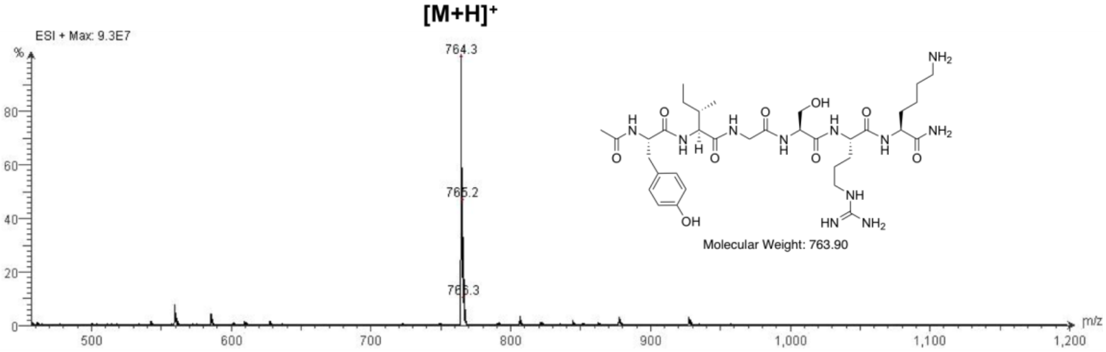

C. $AS_{YIG}RK$

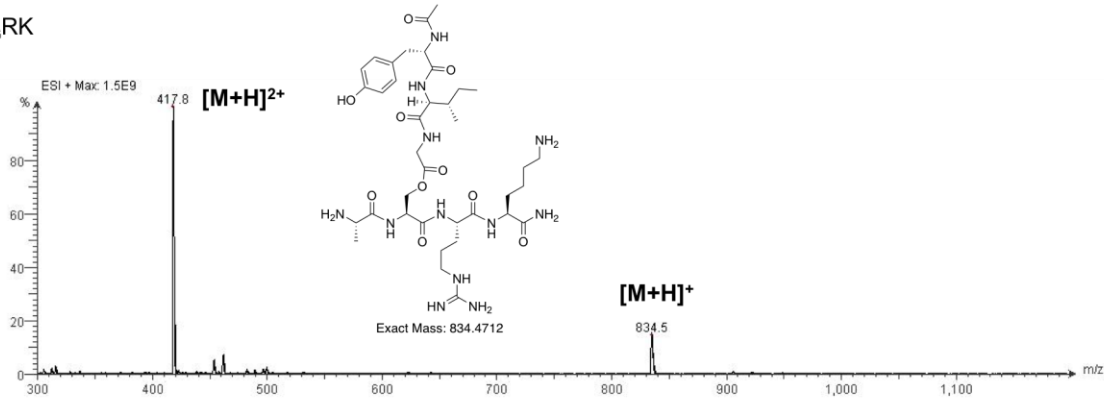

Supplementary Fig. 7. ESI mass spectra of (**A**) $KS_{YIG}RK$; (**B**)YIGSRK; and (**C**) $AS_{YIG}RK$.

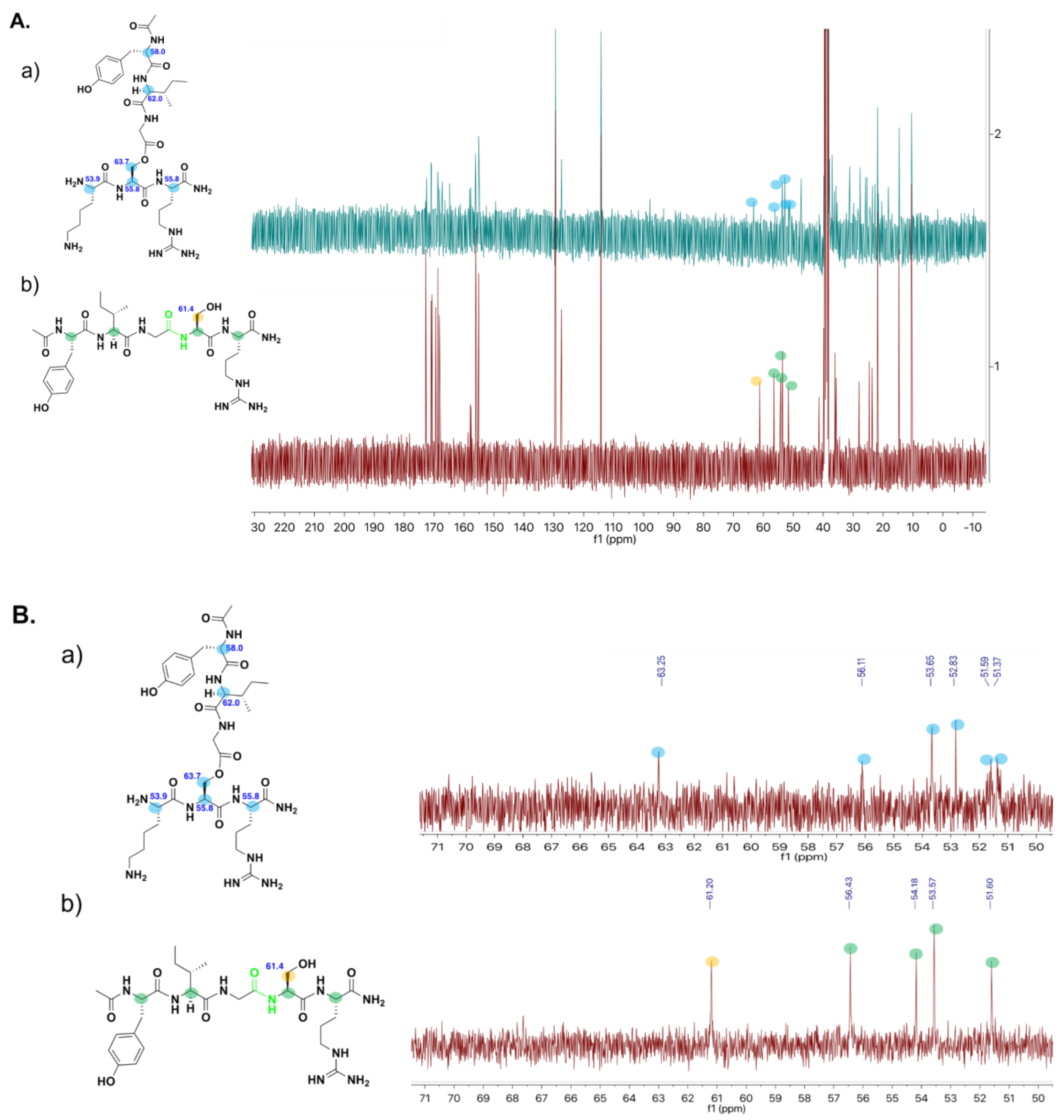

Supplementary Fig. 8. Validation of the switch mechanism. (A) Full and (B) zoomed-in ^{13}C NMR spectra in DMSO-d_6 of a) switch peptide **KS$_{\mathrm{YIG}}$R** and b) functional peptide (after the switch) Ac-YIGSR. The numbers noted on the chemical structures are the chemical shifts predicted by Chemdraw. The 50-70 ppm region was zoomed in to compare the shifts before and after the switch, where only the Ser residue β-carbon shifted.

Cell studies

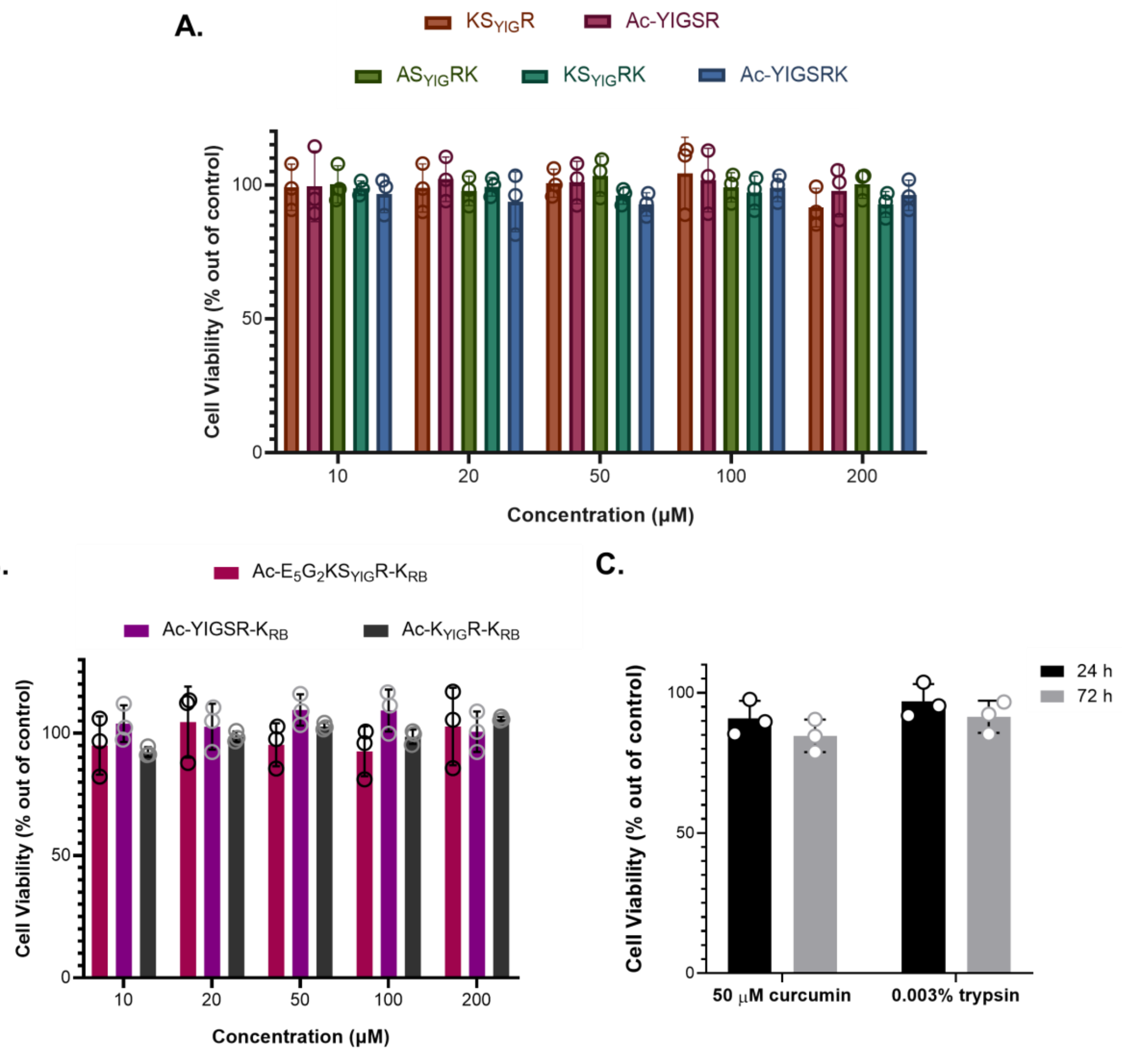

Supplementary Fig. 9. Cell viability of HUVECs treated with **(A)** different concentrations of $KS_{YIG}R$, Ac-YIGSR, $AS_{YIG}RK$, $KS_{YIG}RK$, and Ac-YIGSRK for 24 h; **(B)** different concentrations of rhodamine B-labeled peptides Ac-E_5G_2-$KS_{YIG}R$-K_{RB}, Ac-YIGSR-K_{RB}, and Ac-$K_{YIG}R$-K_{RB}. **(C)** 50 µM curcumin and 0.003% trypsin for 24 h and 72 h. In all cases $n = 3$ independent experiments with 5 replicates per experiment. Data were presented as mean values ± SD.

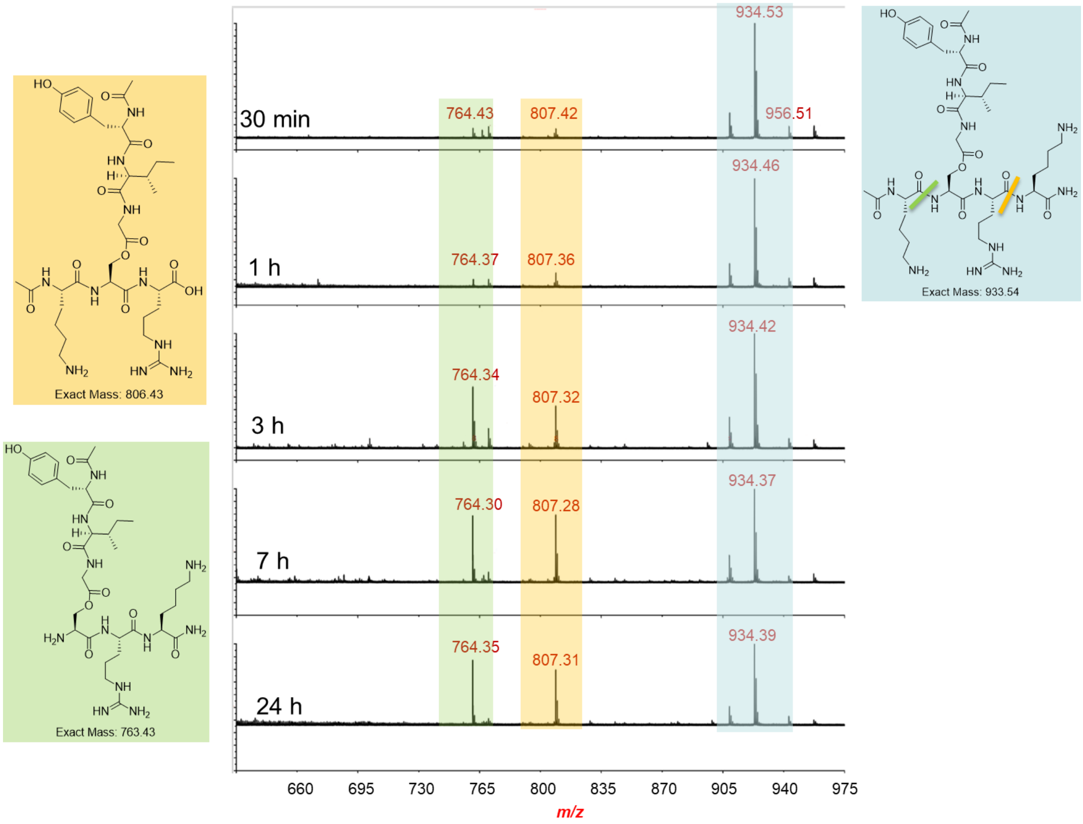

Supplementary Fig. 10. MALDI-TOF spectra of switch peptide Ac-KS_{YIG}RK after incubation with trypsin (0.08 wt% total) in PBS buffer at 37 °C for different time periods. Peak *m*/*z* = 934 (blue box) correlates to the switch peptide. Peak *m*/*z* =807 (yellow box) correlates to product resulting from cleavage on C-terminal side of the Arg residue. Peak *m*/*z* =764 (green box) correlates to the product resulting from cleavage on the N-terminal side of the Lys residue, leading to formation of the functional peptide (the peptide before the O→N acyl shift is shown for sake of clarity, but rapid rearrangement forms the functional peptide).

Supplementary Note 1. Control peptide descriptions:

Ac-E_5G_2-$KS_{YIG}R$-K_{RB} without trypsin treatment: Negative control. To confirm the peptide without rearrangement triggered by trypsin has no cell binding ability, thus will be washed away by PBS and show no fluorescence under the microscope.

Ac- $K_{YIG}R$-K_{RB}: Negative control. To confirm that the fluorescence labeled peptide with YIG on the side chain is not able to attach to the cell membrane and show fluorescence signal under the microscopy.

Ac- $K_{YIG}R$-K_{RB} with trypsin treatment: Negative control. To confirm that the fluorescence labeled peptide with YIG on the side chain is not able to attach to the cell membrane even with the presence with trypsin.

Ac-YIGSR-K_{RB}: Positive control. The functional peptide after rearrangement, indicating the switch peptide after trypsin treatment showed similar fluorescence intensity.

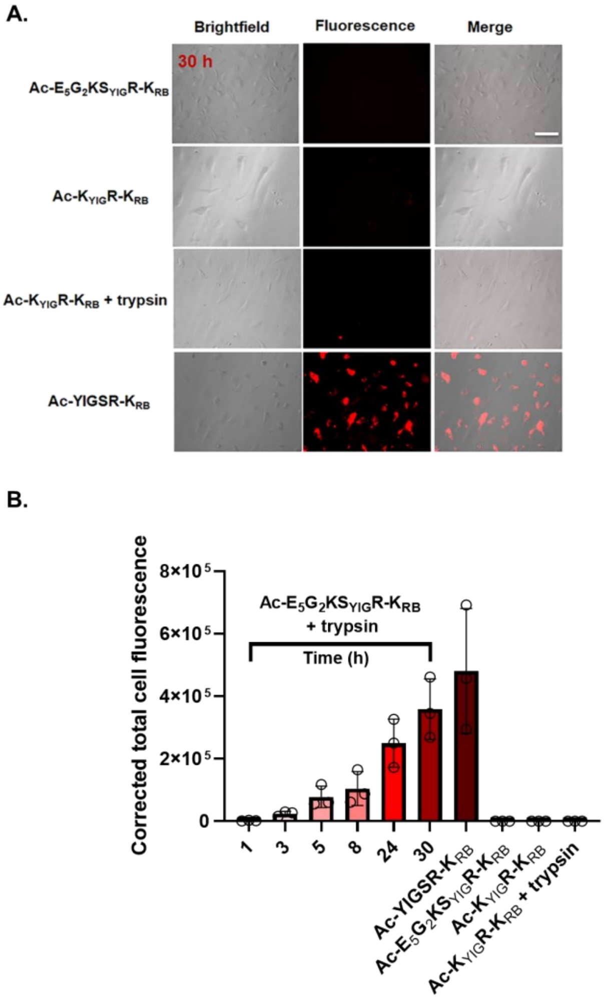

Supplementary Fig. 11. Fluorescence images of control studies in solution. (A) Brightfield, fluorescence, and merged images of HUVECs preincubated with Ac-$E_5G_2KS_{YIG}R$-K_{RB} (final concentration 50 μM), Ac-$K_{YIG}R$-K_{RB} (final concentration 50 μM), Ac-$KS_{YIG}R$-K_{RB} (final concentration 50 μM) with trypsin (0.00025 wt.%), and Ac-YIGSR-K_{RB} (final concentration 50 μM) at 30 h. Scale bar = 100 μm. **(B)** Corrected total cell fluorescence (CTCF) values at each timepoint measured from fluorescence images of Ac-$E_5G_2KS_{YIG}R$-K_{RB} with trypsin, and control peptides mentioned above at 30 h. Average fluorescence intensities were quantified by ImageJ (cell counts are >30 for each group from three separate wells). *n* = 3 independent experiments. Data are presented as mean values ± SD.

The corrected total cell fluorescence (CTCF) was calculated as follows:[1,2]

CTCF = integrated density – (area of selected cell × mean fluorescence of background readings)

Supplementary Table 1. Statistics Data of CTCF at each timepoint measured from fluorescence images of Ac-E$_5$G$_2$KS$_{YIG}$R-K$_{RB}$ with trypsin, and control peptides mentioned above at 30 h.

**Comparison**	**Mean Difference**	**q**	***P* value**
1 h vs 8 h	-102514	na	*ns*
8 h vs 24 h	-145141	3.257	*
24 h vs 30 h	-108676	2.438	*ns*
30 h vs Ac-YIGSR-K$_{RB}$	-122720	2.754	*ns*
30 h vs Ac-E$_5$G$_2$KS$_{YIG}$R-K$_{RB}$	358394	8.042	***
30 h vs Ac-K$_{YIG}$R-K$_{RB}$	358394	8.042	***
30 h vs Ac-K$_{YIG}$R-K$_{RB}$ + trypsin	358396	8.042	***
Ac-YIGSR-K$_{RB}$ vs Ac-E$_5$G$_2$KS$_{YIG}$R-K$_{RB}$	481114	10.80	***
Ac-YIGSR-K$_{RB}$ vs Ac-K$_{YIG}$R-K$_{RB}$	481114	10.80	***
Ac-YIGSR-K$_{RB}$ vs Ac-K$_{YIG}$R-K$_{RB}$ + trypsin	481116	10.80	***
Ac-E$_5$G$_2$KS$_{YIG}$R-K$_{RB}$ vs Ac-K$_{YIG}$R-K$_{RB}$	0.06933	na	*ns*
Ac-E$_5$G$_2$KS$_{YIG}$R-K$_{RB}$ vs Ac-K$_{YIG}$R-K$_{RB}$ + trypsin	1.943	na	*ns*
Ac-K$_{YIG}$R-K$_{RB}$ vs Ac-K$_{YIG}$R-K$_{RB}$ + trypsin	1.873	na	*ns*

Group comparisons are indicated as determined by a one-way analysis of variance (ANOVA) with a Student−Newman−Keuls comparisons post hoc test. *** indicates $p < 0.001$, ** indicates $p < 0.01$, * indicates $p < 0.05$, and *ns* indicates no significance among indicated treatment groups.

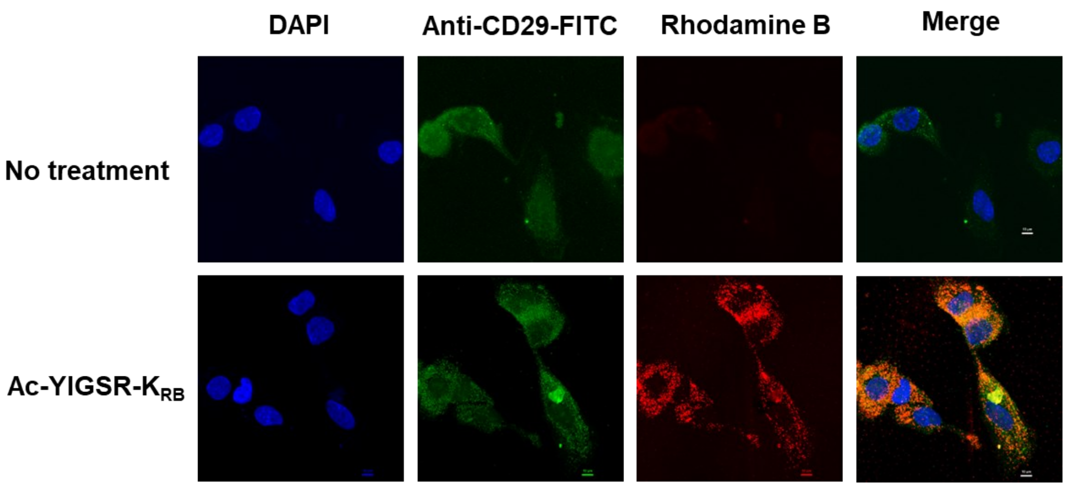

Supplementary Fig. 12. Immunofluorescence staining of adhesion protein CD29 (integrin β1, green) and rhodamine B-labeled peptide Ac-YIGSR-K$_{RB}$ (red) on HUVECs. HUVECs were treated with 50 μM Ac-YIGSR-KRB for 24 h, then cell nuclei were stained with 4,6-diamidino-2-phenylindole (DAPI, blue) and anti-CD29-FITC. Fluorescence microscopy showed that the laminin derived peptide Ac-YIGSR-K$_{RB}$ colocalized with integrin β1 on the cell surface. Scale bar = 10 μm.

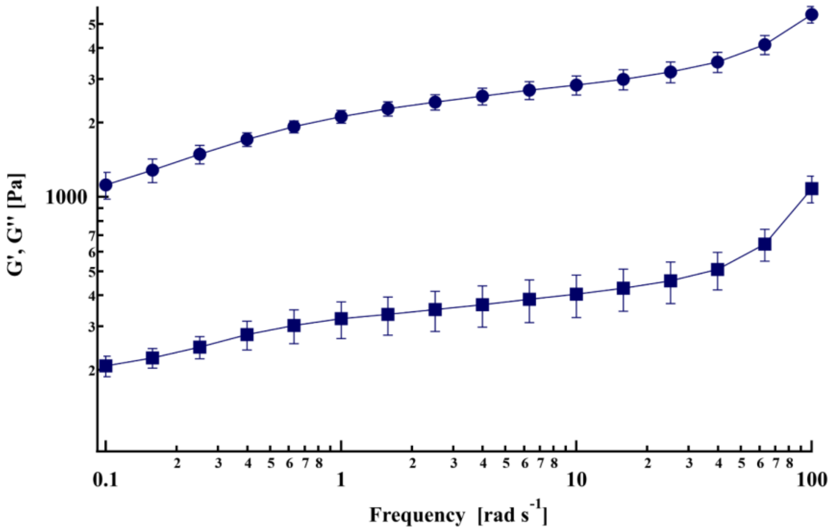

Supplementary Fig. 13. Frequency sweep results of switch peptide-modified alginate hydrogel showing G’ (circles) and G” (squares). Averaged over 3 samples (n = 3). Error bars represent standard deviation.

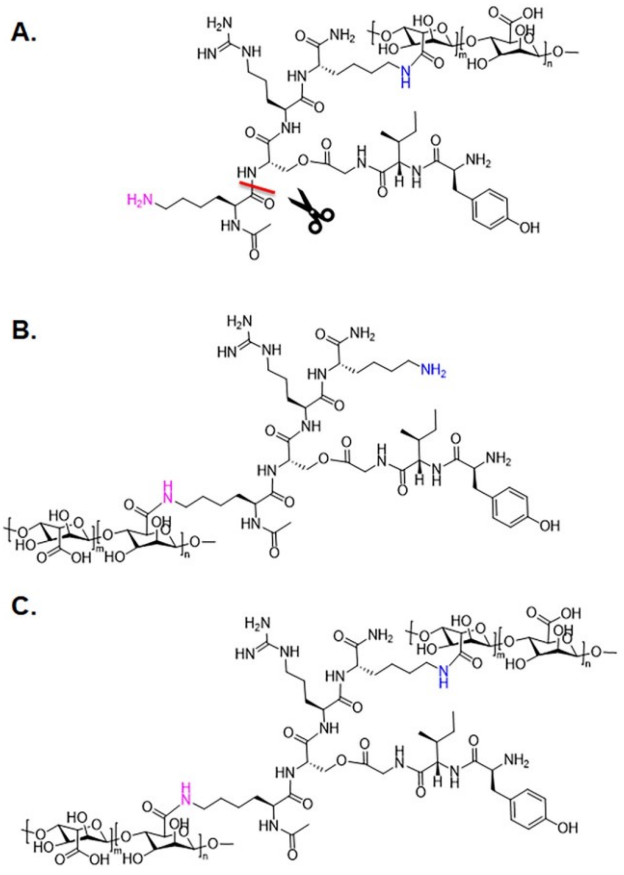

Supplementary Fig. 14. Chemical structures of possible alginate-switch peptides conjugates. (**A**) Alginate conjugated to the ε-amine of the C-terminal Lys (blue). This linkage leaves the ε-amine of the N-terminal Lys (pink) free, which can be recognized by trypsin and thus the Lys can be cleaved and the switch can occur. (**B**) Alginate conjugated to the ε-amine of the N-terminal Lys (pink), but not to the ε-amine of the C-terminal Lys (blue). This linkage inhibits trypsin from recognizing the C-terminus of the Lys residue; thus, the cleavage does not happen and the rearrangement does not occur. (**C**) Alginate conjugated to both amines. In this situation, cleavage cannot occur.

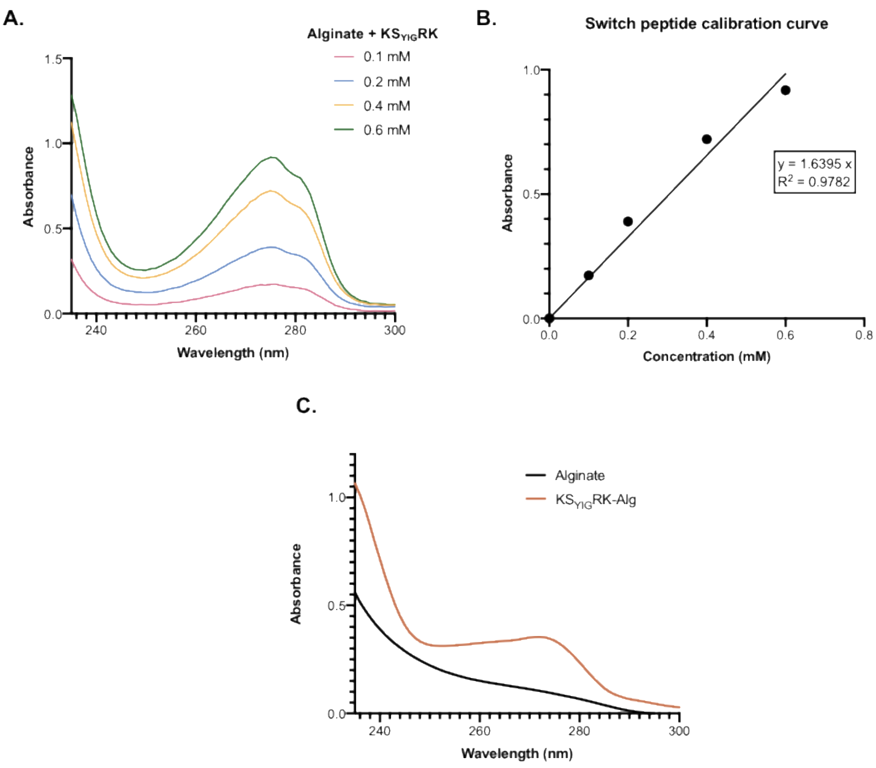

Supplementary Fig. 15. Verification of conjugation of the switch peptide to the alginate backbone. (A) UV-Vis of alginate with different concentrations of switch peptide (KS$_{YIG}$RK) without the addition of coupling reagents. (**B**) Calibration curve of alginate with switch peptide at wavelength 276 nm. The Beer's Law was obtained as y = 1.6395 x, where y stands for absorbance and x stands for peptide concentration (mM). (**C**) UV-Vis of alginate, and KS$_{YIG}$RK-Alg in water after dialysis (original concentrations: KS$_{YIG}$RK-Alg 0.357 mM). The peak at 276 nm corresponds to Tyr residue absorbance. The calculated peptide concentration for switch peptide-modified alginate is 0.208 mM, thus the coupling efficiency is 58% based on the calibration curve.

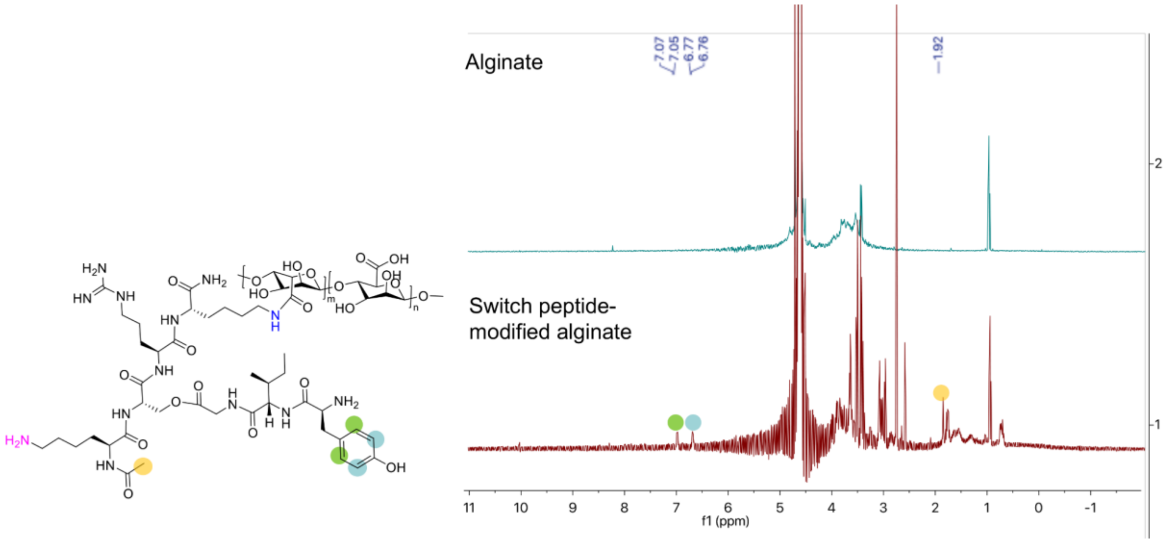

Supplementary Fig. 16. ^{1}H-NMR spectra of alginate (upper panel) and switch peptide-modified alginate after dialysis (bottom panel). Signals appear at chemical shifts consistent with aromatic protons coming from the Tyr amino acid in the switch peptide (6.76 ppm and 7.05 ppm) which are absent in the alginate structure, as well as protons from the acyl group (1.92 ppm). The ^{1}H NMR spectrum of switch peptide-modified alginate was collected at a significantly lower concentration than unmodified alginate (2 versus 10 mg/mL), leading to lower signal intensity.

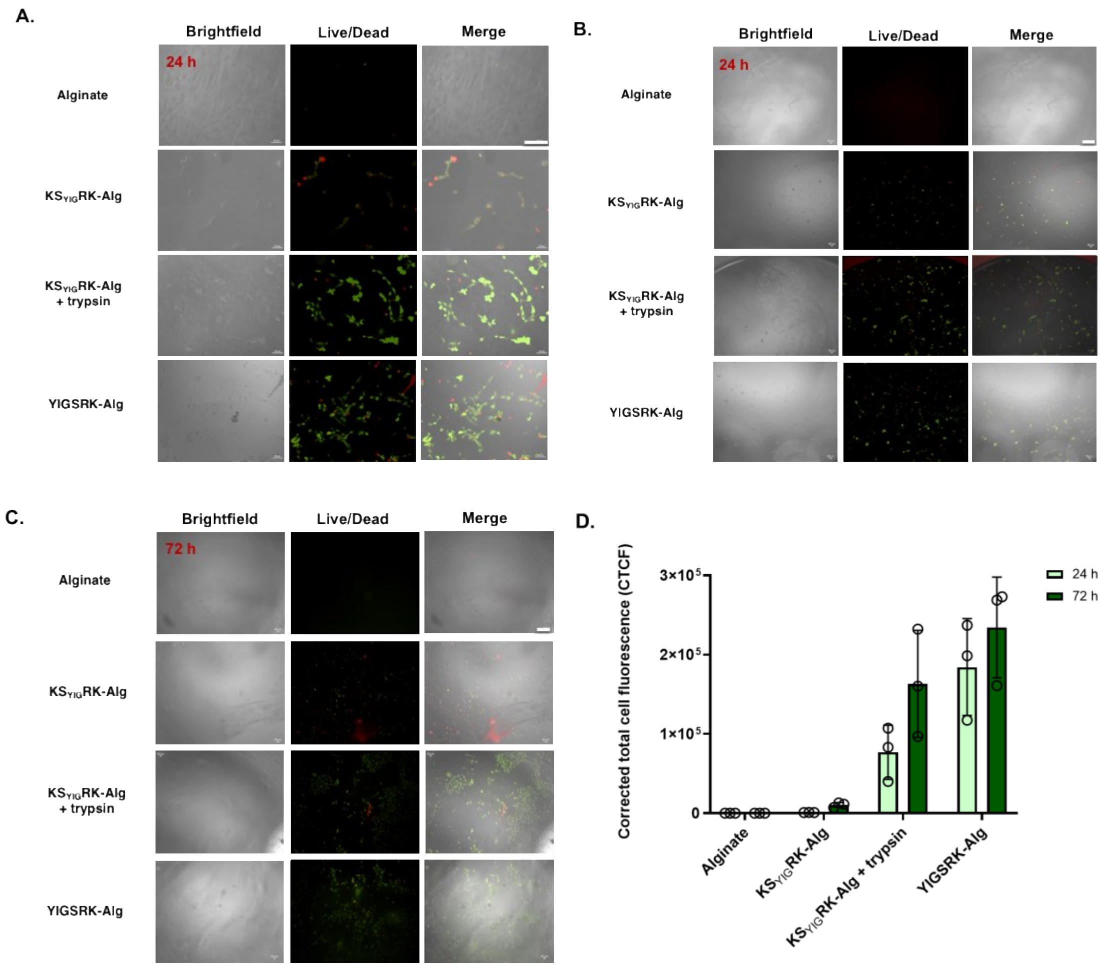

Supplementary Fig. 17. Representative brightfield, live/dead (green/red), and merged images of HUVEC cells adhered to unmodified alginate hydrogel (alginate), switch peptide-modified alginate hydrogel (KS$_{YIG}$RK-Alg), switch peptide-modified alginate hydrogel with 0.05% trypsin added (final concentration 0.003%, KS$_{YIG}$RK-Alg + trypsin), and functional peptide-modified alginate hydrogel (YIGSRK-Alg) for **(A)** 24 h post-seeding. Live/dead staining and subsequent fluorescence microscopy were performed to confirm cell viability and cell spreading. Scale bars represent 200 μm. Magnification = 10×. **(B)** 24 h post-seeding. Live/dead staining and subsequent fluorescence microscopy was performed to confirm cell viability and cell spreading. Scale bars represents 200 μm. Magnification = 4×. **(C)** 72 h post-seeding. Live/dead staining and subsequent fluorescence microscopy was performed to confirm cell viability and cell spreading. Scale bars represents 200 μm. Magnification = 4×. See 72 h post-seeding, magnification = 10× images in **Fig 3D**. **(D).** Corrected total cell fluorescence (CTCF)

values measured from fluorescence images of peptide-alginate hydrogels mentioned above at 24 h and 72 h. Average fluorescence intensities were quantified by ImageJ (cell counts are 30 for each group from three separate wells). *n* = 3 independent experiments. Data are presented as mean values ± SD.

Supplementary Table 2. Statistics Data of CTCF measured from fluorescence images of peptide-alginate hydrogels mentioned above at 24 h and 72 h.

Comparison	Mean Difference	q	*P* value
24 h KSYIGRK-Alg vs 24 h KSYIGRK-Alg+trypsin	-75122	6.679	***
24 h KSYIGRK-Alg vs 24 h YIGSRK-Alg	-184904	17.217	***
24 h KSYIGRK-Alg+trypsin vs 24 h YIGSRK-Alg	-109782	10.222	***
24 h KSYIGRK-Alg+trypsin vs 72 h KSYIGRK-Alg+trypsin	-92959	8.536	***
24 h KSYIGRK-Alg vs 72 h KSYIGRK-Alg	-6657.8	na	*ns*
72 h KSYIGRK-Alg vs 72 h KSYIGRK-Alg+trypsin	-161423	15.987	***
72 h KSYIGRK-Alg vs 72 h YIGSRK-Alg	-213882	22.436	***
72 h KSYIGRK-Alg+trypsin vs 72 h YIGSRK-Alg	-52459	5.256	***
24 h YIGSRK-Alg vs 72 h YIGSRK-Alg	-35636	3.63	*
72 h KSYIGRK-Alg+trypsin vs 24 h YIGSRK-Alg	-16823	1.623	*ns*

Group comparisons are indicated as determined by a one-way analysis of variance (ANOVA) with a Student−Newman−Keuls comparisons post hoc test. *** indicates $p < 0.001$, ** indicates $p < 0.01$, * indicates $p < 0.05$, and *ns* indicates no significance among indicated treatment groups.

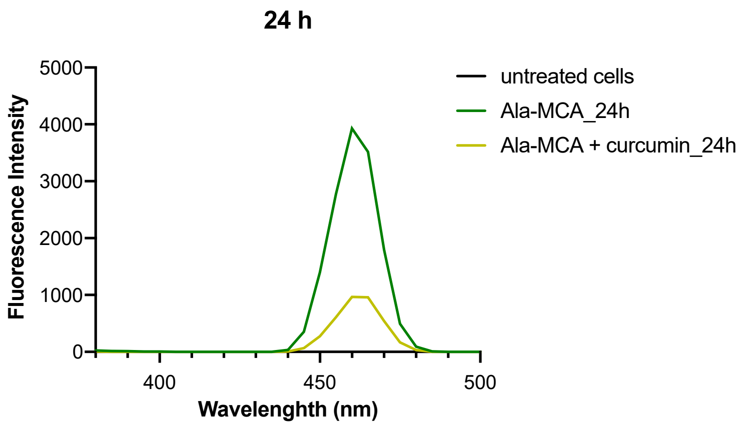

Supplementary Fig. 18. Fluorescence spectroscopy (λ_{ex} = 360 nm) of APN activity with APN substrate Ala-MCA treatment for 24 h and with the addition of APN inhibitor curcumin for 24 h. The APN activity decreased to ~25% with inhibition using curcumin (50 μM).

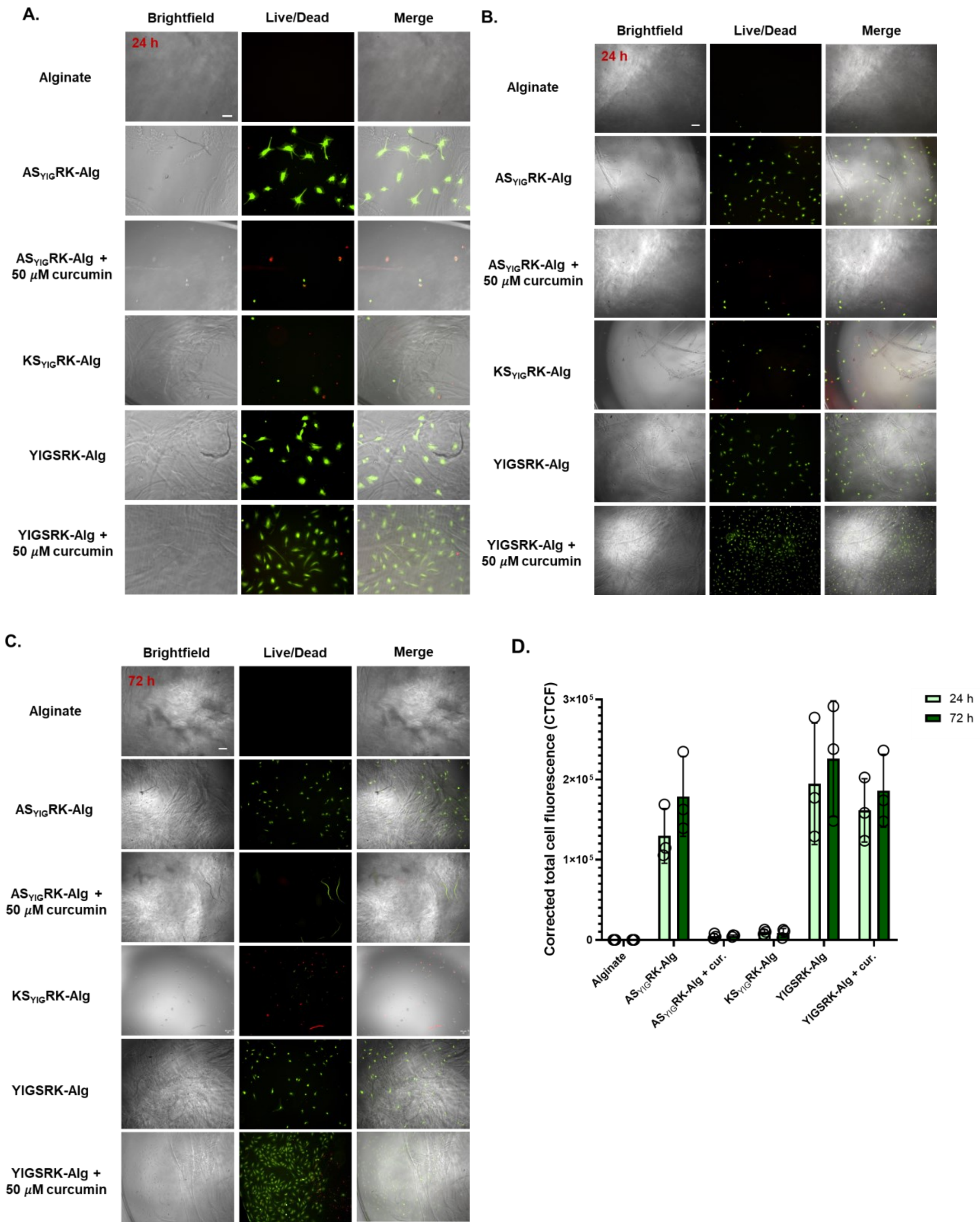
A.
Brightfield
Live/Dead
Merge
24 h
Alginate
AS$_{YIG}$RK-Alg
AS$_{YIG}$RK-Alg + 50 μM curcumin
KS$_{YIG}$RK-Alg
YIGSRK-Alg
YIGSRK-Alg + 50 μM curcumin
B.
Brightfield
Live/Dead
Merge
24 h
Alginate
AS$_{YIG}$RK-Alg
AS$_{YIG}$RK-Alg + 50 μM curcumin
KS$_{YIG}$RK-Alg
YIGSRK-Alg
YIGSRK-Alg + 50 μM curcumin
C.
Brightfield
Live/Dead
Merge
72 h
Alginate
AS$_{YIG}$RK-Alg
AS$_{YIG}$RK-Alg + 50 μM curcumin
KS$_{YIG}$RK-Alg
YIGSRK-Alg
YIGSRK-Alg + 50 μM curcumin
D.
Corrected total cell fluorescence (CTCF)
3×10^5
2×10^5
1×10^5
0
24 h
72 h
Alginate
AS$_{YIG}$RK-Alg
AS$_{YIG}$RK-Alg + cur.
KS$_{YIG}$RK-Alg
YIGSRK-Alg
YIGSRK-Alg + cur.

Supplementary Fig. 19. Representative brightfield, live/dead (green/red), and merged images of HUVEC cells adhered to unmodified alginate hydrogel, AS$_{\text{YIG}}$RK-modified alginate hydrogel, AS$_{\text{YIG}}$RK-modified alginate hydrogel with curcumin (50 μM) added, switch peptide-modified alginate hydrogel, functional peptide-modified alginate hydrogel, and YIGSRK-modified alginate hydrogel with curcumin (50 μM) added for (**A**) 24 h post-seeding. Live/dead staining and subsequent fluorescence microscopy were performed to confirm cell viability and cell spreading. Scale bar represents 100 μm. Magnification = 10×. (**B)** 24 h post-seeding. Live/dead staining and subsequent fluorescence microscopy were performed to confirm cell viability and cell spreading. Scale bar represents 200 μm. Magnification = 4×. **(C)** 72 h post-seeding. Live/dead staining and subsequent fluorescence microscopy were performed to confirm cell viability and cell spreading. Scale bar represents 200 μm. Magnification = 4×. See 72 h post-seeding, magnification = 10× images in **Fig 3F**. (**D)** Corrected total cell fluorescence (CTCF) values measured from fluorescence images of peptide-alginate hydrogels mentioned above at 24 h and 72 h. Average fluorescence intensities were quantified by ImageJ (cell counts are 30 for each group from three separate wells). n = 3 independent experiments. Data are presented as mean values ± SD.

Supplementary Table 3. Statistics Data of CTCF measured from fluorescence images of peptide-alginate hydrogels mentioned above at 24 h and 72 h.

Comparison	Mean Difference	q	*P* value
24 h AS$_{YIG}$RK-Alg vs 72 h AS$_{YIG}$RK-Alg	-49904	4.497	**
24 h AS$_{YIG}$RK-Alg vs 24 h AS$_{YIG}$RK-Alg + cur.	-172006	13.662	***
24 h AS$_{YIG}$RK-Alg vs 24 h KS$_{YIG}$RK-Alg	-117751	10.433	***
24 h AS$_{YIG}$RK-Alg vs 24 h YIGSRK-Alg	-58276	5.656	***
24 h AS$_{YIG}$RK-Alg + cur. vs 24 h KS$_{YIG}$RK-Alg	-4349.9	na	*ns*
24 h AS$_{YIG}$RK-Alg + cur. vs 24 h YIGSRK-Alg	-180377	15.162	***
24 h AS$_{YIG}$RK-Alg + cur. vs 72 h AS$_{YIG}$RK-Alg + cur.	-682.01	na	*ns*
24 h KS$_{YIG}$RK-Alg vs 24 h YIGSRK-Alg	-176027	17.085	***
24 h KS$_{YIG}$RK-Alg vs 72 h KS$_{YIG}$RK-Alg	-401.37	na	*ns*
24 h YIGSRK-Alg vs 72 h YIGSRK-Alg	-34586	3.596	*
72 h AS$_{YIG}$RK-Alg vs 72 h AS$_{YIG}$RK-Alg + cur.	-172688	14.166	***
72 h AS$_{YIG}$RK-Alg vs 72 h KS$_{YIG}$RK-Alg	-167254	15.339	***
72 h AS$_{YIG}$RK-Alg vs 72 h YIGSRK-Alg	-47610	4.561	**
72 h AS$_{YIG}$RK-Alg + cur. vs 72 h KS$_{YIG}$RK-Alg	-5433.3	na	*ns*
72 h AS$_{YIG}$RK-Alg + cur. vs 72 h YIGSRK-Alg	-220298	18.705	***
72 h KS$_{YIG}$RK-Alg vs 72 h YIGSRK-Alg	-214865	20.582	***
72 h AS$_{YIG}$RK-Alg vs 24 h YIGSRK-Alg	-8371.6	0.8293	*ns*
24 h AS$_{YIG}$RK-Alg vs 24 h YIGSRK-Alg + cur.	-39800	3.940	**
24 h YIGSRK-Alg + cur. vs 72 h YIGSRK-Alg + cur.	-16805	na	*ns*
24 h YIGSRK-Alg + cur. vs 24 h YIGSRK-Alg	-23128	2.615	*ns*
72 h AS$_{YIG}$RK-Alg vs 72 h YIGSRK-Alg + cur.	-6700.2	na	*ns*
72 h YIGSRK-Alg + cur. vs 72 h YIGSRK-Alg	-40910	4.373	**

Group comparisons are indicated as determined by a one-way analysis of variance (ANOVA) with a Student−Newman−Keuls comparisons post hoc test. *** indicates $p < 0.001$, ** indicates $p < 0.01$, * indicates $p < 0.05$, and *ns* indicates no significance among indicated treatment groups.

Supplementary References:

1 McCloy, R. A. *et al.* Partial inhibition of Cdk1 in G2 phase overrides the SAC and decouples mitotic events. *Cell cycle* **13**, 1400-1412 (2014).

2 Schindelin, J. *et al.* Fiji: an open-source platform for biological-image analysis. *Nat. methods* **9**, 676-682 (2012).
