# Supplementary material for: Activating hidden signals by mimicking cryptic sites in a synthetic extracellular matrix
Source: Nat Commun. 2023 Jun 19;14:3635. doi: 10.1038/s41467-023-39349-w (PMC10279755; doi:10.1038/s41467-023-39349-w)
Supplement: Supplementary file 3 — Reporting Summary [file 41467_2023_39349_MOESM3_ESM.pdf]

nature portfolio

Corresponding author(s): Matson, Bitton

Last updated by author(s): 11May2023

Reporting Summary

Nature Portfolio wishes to improve the reproducibility of the work that we publish. This form provides structure for consistency and transparency in reporting. For further information on Nature Portfolio policies, see our Editorial Policies and the Editorial Policy Checklist.

Statistics

For all statistical analyses, confirm that the following items are present in the figure legend, table legend, main text, or Methods section.

n/a	Confirmed	
☐	☒	The exact sample size (*n*) for each experimental group/condition, given as a discrete number and unit of measurement
☐	☒	A statement on whether measurements were taken from distinct samples or whether the same sample was measured repeatedly
☐	☒	The statistical test(s) used AND whether they are one- or two-sided<br>*Only common tests should be described solely by name; describe more complex techniques in the Methods section.*
☐	☒	A description of all covariates tested
☐	☒	A description of any assumptions or corrections, such as tests of normality and adjustment for multiple comparisons
☐	☒	A full description of the statistical parameters including central tendency (e.g. means) or other basic estimates (e.g. regression coefficient) AND variation (e.g. standard deviation) or associated estimates of uncertainty (e.g. confidence intervals)
☐	☒	For null hypothesis testing, the test statistic (e.g. F, t, r) with confidence intervals, effect sizes, degrees of freedom and P value noted<br>*Give P values as exact values whenever suitable.*
☒	☐	For Bayesian analysis, information on the choice of priors and Markov chain Monte Carlo settings
☒	☐	For hierarchical and complex designs, identification of the appropriate level for tests and full reporting of outcomes
☒	☐	Estimates of effect sizes (e.g. Cohen's d, Pearson's r), indicating how they were calculated

Our web collection on statistics for biologists contains articles on many of the points above.

Software and code

Policy information about availability of computer code

Data collection

NMR spectroscopy - Bruker Topspin 3.2 pl7 (patch level)
Fluorescence Microscope-NIS-Elements AR (Basic Research) version 3.22.00
Confocal Microscope - NIS-Elements AR (Advanced Research) version 5.02.00
SEM - Microscope control version 5.5.2 build 3322
Rheometer - Anton Paar RheoCompass version 1.30
MALDI- ABSciex 4000 Series Explorer version 3.0
UV-vis - Varian Cary WinUV version 4.10(464)
Fluorimeter - BioTek Gen5 version 2.05.5

Data analysis

NMR spectroscopy - MestReNova version 14.2.1-27684
Fluorescence Microscope - NIS-Elements AR (Basic Research) version 3.22.00
Confocal Microscope - NIS-Elements AR (Advanced Research) version 5.02.00
SEM - Microscope control v5.5.2 build 3322
Rheometer - Anton Paar RheoCompass version 1.30
MALDI-TOF mass spectrometry - ABSciex 4000 Series Explorer™ Software version 3.0
CTCF - ImageJ version 1.53t
Fluorimeter - Biotek Gen5 version 2.05.5
ChemDraw - version 20.0.0.41
GraphPad Prism 8.0 for statistics

For manuscripts utilizing custom algorithms or software that are central to the research but not yet described in published literature, software must be made available to editors and reviewers. We strongly encourage code deposition in a community repository (e.g. GitHub). See the Nature Portfolio guidelines for submitting code & software for further information.

Data

Policy information about availability of data

All manuscripts must include a data availability statement. This statement should provide the following information, where applicable:

- Accession codes, unique identifiers, or web links for publicly available datasets
- A description of any restrictions on data availability
- For clinical datasets or third party data, please ensure that the statement adheres to our policy

Source data are provided as a Source Data file

Human research participants

Policy information about studies involving human research participants and Sex and Gender in Research.

Reporting on sex and gender: N/A

Population characteristics: N/A

Recruitment: N/A

Ethics oversight: N/A

Note that full information on the approval of the study protocol must also be provided in the manuscript.

Field-specific reporting

Please select the one below that is the best fit for your research. If you are not sure, read the appropriate sections before making your selection.

☒ Life sciences ☐ Behavioural & social sciences ☐ Ecological, evolutionary & environmental sciences

For a reference copy of the document with all sections, see nature.com/documents/nr-reporting-summary-flat.pdf

Life sciences study design

All studies must disclose on these points even when the disclosure is negative.

Sample size: Sample sizes were 5 wells (replicates) per experiment in the cell studies. No statistical methods were used to pre-determine sample size. This sample sizes are common in cell studies and are sufficient for scatter in in vitro live/dead studies.

Data exclusions: No data were excluded from the study.

Replication: For cell viability assay, mean values are reported together with the standard error of the mean (SEM) representing the combination of 3 independent experimental runs with five replicates per experiment. All attempts at replication were successful.

For fluorescence study, mean fluorescence intensity of the background was calculated by averaging the intensities of selected areas that had no fluorescence. Cell counts were >30 for each group from three separate wells.

Randomization: Deliberate randomization was not relevant to this cell work because cells were homogenously suspended before plating.

Blinding: Blinding was not relevant to this study because these cell studies required simple, predetermined treatments.

Reporting for specific materials, systems and methods

We require information from authors about some types of materials, experimental systems and methods used in many studies. Here, indicate whether each material, system or method listed is relevant to your study. If you are not sure if a list item applies to your research, read the appropriate section before selecting a response.

Materials & experimental systems

n/a	Involved in the study
☐	☒ Antibodies
☐	☒ Eukaryotic cell lines
☒	☐ Palaeontology and archaeology
☒	☐ Animals and other organisms
☒	☐ Clinical data
☒	☐ Dual use research of concern

Methods

n/a	Involved in the study
☒	☐ ChIP-seq
☒	☐ Flow cytometry
☒	☐ MRI-based neuroimaging

Antibodies

Antibodies used

Anti-CD29 Armenian Hamster Monoclonal Antibody (FITC (Fluorescein Isothiocyanate)).
Supplier - BioLegend
Cat # - 102205
Clone - HMbeta1-1
Lot # - B348691

Validation

According to the manufacturer, each lot of this antibody is quality control tested by immunofluorescent staining with flow cytometric analysis.

Eukaryotic cell lines

Policy information about cell lines and Sex and Gender in Research

Cell line source(s)

Human Umbilical Vein Endothelial Cells (HUVECs)

Authentication

The HUVECs were authenticated by immunostaining for CD31 and vWF

Mycoplasma contamination

The HUVECs are tested routinely for mycoplasma contamination and found to be negative

Commonly misidentified lines
(See ICLAC register)

N/A
